# A randomized two way cross over study for comparison of absorption of vitamin D3 buccal spray and soft gelatin capsule formulation in healthy subjects and in patients with intestinal malabsorption

**DOI:** 10.1186/s12937-015-0105-1

**Published:** 2015-10-29

**Authors:** MC Satia, AG Mukim, KD Tibrewala, MS Bhavsar

**Affiliations:** 1 Ethicare Clinical Trial Services, Ahmedabad, India; 2 Mukim Medical And Nursing Homes, Ahmedabad, India; 3 Tibrewala’s Clinic, Ahmedabad, India; 4 Bhavsar’s Clinic, Ahmedabad, India

**Keywords:** Vitamin D3, 25-hydroxyvitamin D, Malabsorption Syndrome, Buccal Spray, Cross Over Study

## Abstract

**Background:**

Vitamin D deficiency has been proposed to contribute to the development of malabsorption diseases. Despite this, the vitamin D status of these patients is often neglected. The objective of the present work was to compare the absorption of vitamin D_3_ through the oral route by comparing a 1000 IU soft gelatin capsule and a 500 IU buccal spray (delivering 1000 IU in two spray shots) in healthy subjects and in patients with malabsorption disease.

**Methods:**

An open label, randomized, two-periods, two-way cross over study was conducted, first in healthy subjects (n = 20) and then in patients with malabsorption syndrome (n = 20). The study participants were equally divided and received either of the treatments (buccal spray, n = 7; soft gelatin capsule, n = 7; control, n = 6) in Period I for 30 days. After washout of another 30 days, the treatments were changed in crossover fashion in Period II. Fasting blood samples were collected to measure baseline 25-hydroxyvitamin D [25(OH)D] levels in all participants at day 0 (Screening visit), day 30 (completion of period I), day 60 (end of wash out and initiation of period II) and day 90 (completion of period II). Safety was evaluated by hematology and biochemistry analyses. Statistical analyses was performed using differences of mean and percentage change from baseline of 25(OH)D levels between two formulation by two tailed Paired t-test with 95 % confidence interval.

**Results:**

In healthy subjects, the mean increase in serum 25(OH)D concentration was 4.06 (95 % CI 3.41, 4.71) ng/ml in soft gelatin capsule group and 8.0 (95 % CI 6.86, 9.13) ng/ml in buccal spray group after 30 days treatment (p < 0.0001). In patients with malabsorption disease, the mean increase in serum 25(OH)D concentration was 3.96 (95 % CI 2.37, 5.56) ng/ml in soft gelatin capsule group and 10.46 (95 % CI 6.89, 14.03) ng/ml in buccal spray group (p < 0.0001).

**Conclusion:**

It can be concluded from the results that the buccal spray produced a significantly higher mean serum 25(OH)D concentration as compared to the soft gelatin capsule, in both healthy subjects as well as in patients with malabsorption syndrome over a period of 30 days administration in a two way cross over study. Treatments were well tolerated by both subject groups

**Trial Registration:**

CTRI/2013/06/003770

## Introduction

Vitamin D is essential for active intestinal calcium absorption and plays a central role in maintaining calcium homeostasis and skeletal integrity. It is derived mainly from cutaneous synthesis in the presence of ultraviolet sunlight while dietary intake constitutes a minor fraction [1]. Vitamin D deficiency is a common problem through the world [2, 3] and is assessed by low serum concentration of the major circulating metabolite 25-hydroxyvitamin D (25(OH)D) [4, 5]. The prevention of vitamin D deficiency and insufficiency remains a priority of international health services [6–8]. Vitamin D deficiency has been proposed to contribute to the development of intestinal bowel diseases like Crohn's disease, steatorrhea and ulcerative colitis [9]. Conversely, people who have such illnesses have a reduced absorption of vitamin D3 through the intestine [10, 11]. In addition, osteomalacia occurs in patients with a wide variety of disorders affecting the stomach and small bowel, especially when associated with steatorrhea. The pathogenesis of this osteomalacia has in part been explained by malabsorption in vitamin D [9]. Earlier reports show that orally administered tritiated vitamin D_3_ was malabsorbed in patients with celiac disease, biliary obstruction or pancreatic disease [12]. The pathogenesis of vitamin D deficiency in these patients remains unclear but it is thought to result primarily from fat soluble vitamin malabsorption due to the presence of intestinal disease conditions

Despite vitamin D malabsorption in patients with gastrointestinal or liver disease, the vitamin status of these patients is often neglected. Although vitamin D supplements are often prescribed, adequate absorption of these formulations has not been documented [10]. Vitamin D is known to be liposoluble, and its relative bioavailability could result in unfavorable conditions when administered in solid form (capsule), since the process of its release is a factor limiting the rate of absorption, bearing in mind that bioavailability is related not only to the pharmaceutically active molecules, but also, to the formulation and excipients used.

Vitamin D_3_ taken by oral route (peroral delivery) is absorbed in the intestine, where the lining of the digestive tract is aqueous in nature. Therefore vitamin D_3_, a fat-soluble molecule, in order to be absorbed, must be made water soluble in the intestine. This is accomplished in two steps: emulsification of vitamin D_3_ in the intestinal lumen, through the action of bile salts, forming small droplets which are dispersed and incorporated into micelles-complex aggregates formed by the interaction of free fatty acids, monoglycerides, and bile salts. Micelles are sufficiently water-soluble to access the intestinal brush border where upon the vitamin D_3_ content is released and then absorbed [13].

When sprayed inside the mouth, the fine micro sized droplets of vitamin D_3_ are believed to be quickly and completely absorbed through the buccal mucosa into the numerous capillaries and veins lying close to the tissue surface [13]. Considering the possibility of reduced vitamin D absorption in healthy subjects and even in patients with malabsorption syndrome, a buccal spray formulation was developed. Therefore, the objective of the present work was to compare the absorption of vitamin D_3_ through the oral route (soft gelatin capsule form) and buccal spray in healthy subjects and patients with intestinal malabsorption syndrome.

## Methods

### Study design and patients

An open label, randomized, two-periods, two-way cross over study was conducted, first in healthy subjects and then in patients with malabsorption syndrome, with a similar study design except the presence of disease status in patients with malabsorption syndrome. After approval from the Spandan-IEC ethics committee (registration no: ECR/67/Indt/GJ/2013), the informed consent of study participants were taken and the formulation was administered for 30 days in period I where half of the subjects and patients (collectively participants) received capsule formulation and half of the participants received buccal spray formulation. After completion of treatment in period I, all participants were given 30 days wash out before initiating period II where treatment has changed in cross over fashion, i.e. the participants who received capsule formulation in period I have received buccal spray in period II and vice versa. The clinical study was registered in a centralized clinical trial registry of India (CTRI) before initiating the enrollment of the first patient in the study (CTRI/2013/06/003770).

The inclusion criteria were as follows: subjects of either sex between 18 and 65 years of age, with a Body Mass Index (BMI) between 18.0 and 30.0 kg/m^2^, and the ability to comply with study procedures in the opinion of the investigators. For healthy subjects: no history of liver, kidney or cardiovascular disease, or of any other medical conditions or medications likely to affect vitamin D_3_ absorption or metabolism. For patients with malabsorption syndrome: confirmed diagnosis of any one of the following malabsorption disease conditions like ulcerative colitis, Crohn’s disease or steatorrhea. Patient with history of above diseases, who are on therapy, were selected for the screening. In these patients, malabsorption syndrome was diagnosed by clinical symptoms like abdominal pain, vomiting, diarrhea, and subcutaneous fat loss together with blood tests like haematology and biochemistry. Stool examination was also performed for all patients to confirm rectal bleeding, presence of occult blood, infectious organisms, or fat. Finally, colonoscopy was performed in all patients to objectively assess the extent of inflammation to confirm the diagnosis. The exclusion criteria were as follows: systemic inflammatory or malignant disease; hepatic or renal failure; uncontrolled hypo- or hyperthyroidism, or the use of drugs that are known to affect bone metabolism such as bisphosphonates, glucocorticoids and anti-convulsants. A pregnant or desired to be pregnant woman during study period was also excluded.

### Data collected at baseline

Each participant completed a self-administered questionnaire before enrollment. During completion of the questionnaire, they had the possibility to ask for assistance (i.e. clarification of question or any other issue) from one of the project leaders. The questionnaire included questions about usual intake of vitamin D containing foods, clothing and sun exposure habits, as well as height & weight, date of birth, and education. Body mass index was derived as wt/ht^2^ (kg/m^2^). Blood pressure was measured through sphygmomanometer and vitals (heart rate, body temperature) were also recorded for safety purposes.

### Collection and analyses of blood samples

Fasting blood samples were collected to measure baseline 25(OH)D levels in all the participants at day 0 (Screening visit), day 30 (completion of period I), day 60 (end of wash out and initiation of period II) and day 90 (completion of period II). Blood samples were centrifuged (15 min; 2000 g at 4 °C) within 30 min of blood collection and separated serum samples were immediately frozen. Serum samples were stored at −20 °C until analyzed. Serum 25(OH)D levels were measured by Electrochemiluminescence (ECLIA) assay method. This assay was carried out through quantitative determinations of total 25-hydroxyvitamin D in serum samples using a standard kit available from Roche diagnostics GmbH, Germany. All analyses were done in a central independent clinical analysis laboratory (APL Institute of Clinical Laboratory & Research Pvt. Ltd., Ahmedabad, India). The kit has a limit of detection of 3 ng/mL and has a linearity of 0.0 to 60.0 ng/mL. The intra-assay and inter-assay co-efficient of variation were 4 % and 6 %, respectively. Elecsys e-immunoassay analyzers were used for this assay.

Safety parameters were evaluated including hematology analyses (complete blood counts), biochemical analyses (serum creatinine, total bilirubin, urea, SGOT, SGPT, alkaline phosphates, calcium) and urine was collected for urine routine and microbiological analyses at screening visit (day 0) and at the end of period II visit (day 90).

## Intervention randomization and compliance

### Randomization and group allocation:

The participants were enrolled at two different hospital sites in India; one physician’s site where all healthy subjects were recruited and a gastroenterologist’s site where all patients with intestinal malabsorption were recruited. Out of the forty-eight participants who met the eligibility criteria, forty (twenty healthy and twenty patients) had agreed to participate and were found eligible, signed a written consent form, and completed a self administered questionnaire concerning usual diet and sun exposure. Subsequently, a venous blood sample was drawn, and a participant received a sealed, non-transparent envelope with the allocated treatment intervention. The randomization procedure was performed beforehand by a statistician by block randomization with blocks of two to one ratio, where the first two participants were randomly given each treatment and a third participant served as a control and didn’t receive any treatment. The participants were allocated to the interventions as they visited the clinic i.e. the first subject was allocated to buccal spray group, second to soft gelatin capsule group and third to the control group. This was done in order to distribute the participants equally in the two interventions (Group I and Group III) and half of the participants served as a control (Group II and Group IV).

Twenty healthy subjects enrolled for the study were recruited at a physician’s site. They were randomized and enrolled into group I and II: fourteen subjects were enrolled for vitamin D3 treatment and labeled as group I (healthy subjects), while every third subject (total six subjects) were not given any treatment (labeled as group II) and acted as the control for group I.

The twenty patients with confirmed diagnosis of malabsorption syndromes (nine with ulcerative colitis, four with Crohn’s disease and seven with steatorrhea) were recruited at a gastroenterologist’s hospital site. Fourteen subjects were enrolled for vitamin D_3_ treatment and labeled as group III (patients with malabsorption syndrome), while every third subject (total six subjects) were not given any treatment (labeled as group IV) and acted as the control for group III. All patients continued to take their treatment for ulcerative colitis, Crohn’s disease and steatorrhea as prescribed by gastroenterologists.

Treatment allocation was assigned to each participant according to their number of sequence of attendance at the blood sampling. Treatment allocation was concealed in envelopes numbered in ascending order that they met. Study personnel involved in recruitment and data collection were blinded to the participant’s treatment allocation. All participants were instructed to maintain their routine lifestyle including diet habits and sun exposure during the entire study period, to minimize interference with the daily routine.

### Intervention

The buccal spray and soft gelatin capsules containing vitamin D_3_ were supplied by Pharma Base, India (a subsidiary of Pharma Base SA, Switzerland). The soft gelatin capsule formulation was purchased from the Indian market. The analysis of both the formulations was done in triplicate according to the method described in European Pharmacopeia at an independent analytical laboratory (Oasis Testing House, Ahmedabad, India). The soft gelatin capsule formulation had a label content of 1000 IU per capsule and the buccal spray formulation had a label content of 500 IU/spray shot.

**Fig. 1:**
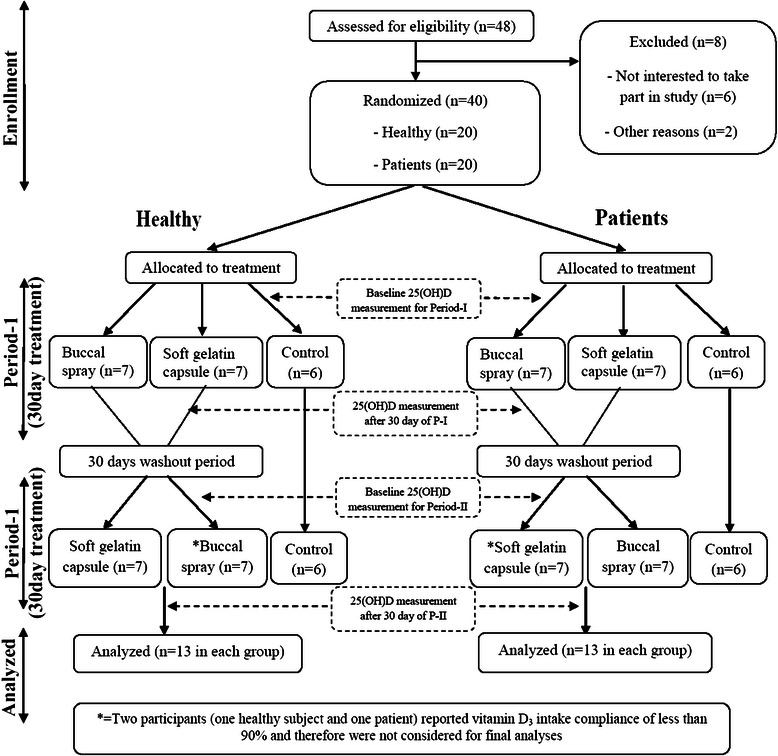
Study flow chart

All participants in group I & III were randomized to receive either the vitamin D_3_ buccal spray (2 sprays each of 500 IU) or soft gelatin capsule containing vitamin D_3_ (1000 IU) for 30 days. Of the fourteen subjects in each group, half received buccal spray and half received soft gelatin capsule. This was considered as period I of the study. After the completion of the 30-day treatment, all participants were given a 30-day washout. The next treatment in period II was changed in a crossover fashion; those participants who had received the buccal spray formulation (vitamin D3) in period I received the soft gelatin capsule formulation in period II and vice versa. The treatment in period II continued in group I and group III participants for the next 30 days. The detailed study flow chart is described in Fig. 1.

### Compliance

All participants were instructed to take the two buccal sprays and one soft gelatin capsule every day irrespective of the period of the study. Formulation for 7 days was handed over to each participants under group I & III at baseline, along with the compliance form. The participants were instructed to mark the intake and time of intake of number of spray shots or capsule of each day of the study period, as well as to note any extraordinary event that occurred during the period (forgetting to take a spray shot or capsule). If forgetting to take spray shot or capsule, the participants were instructed to take a double dose on the following day, in order to take altogether 60 spray shots or 30 capsules during the study periods. The participants were recommended to take spray shots or capsules after the main meal of the day. However, participants were not instructed to standardize meals or time between meals. All participants were also instructed not to change their daily routine, meal habits, as well as their sun exposure. This was done to minimize interference with daily routine and thus maximizing compliance with taking study medication. All participants in group I and III were instructed to visit the site every week to check compliance with the intake of study medication. Participants in control groups (Group II and Group IV) were instructed to visit their respective clinic after 30 days for their blood sample collection.

All participants who received treatment were also instructed to note any adverse events during the entire 90 days of the study which included period I, washout period and period II in a separately given adverse event recording form.

## Stastical Analysis

### Sample size

The sample size of this crossover study was based on the changes in 25(OH)D levels where goal was to detect: 1) percent increase between pre-dose and post-dose levels within treatments, and 2) to have significant difference of percent change in 25(OH)D levels between the treatments. To achieve type-I error rate of less than 5 % (2 tailed), a sample size of 12 subjects in buccal spray group (healthy or patients) was sufficient to provide a statistical power of 80 % to detect a clinically significant mean difference of 5 % in 25(OH)D levels.

### Demographic and baseline characteristic

A descriptive statistics was applied for demographics; age (years), weight (kg), body mass index (kg/m^2^); and were presented as mean ± standard deviation.

### Efficacy analysis

Statistical analyses were performed using GraphPad Prism 5, version 5.03 (GraphPad Software, Inc., CA, USA). Differences of mean and percentage change from baseline of 25(OH)D levels between two formulation groups, soft gelatin capsules and buccal spray, were evaluated using two tailed Paired t-test with 95 % confidence interval separately for both healthy subjects and patients with intestinal malabsorption. For comparison between control group and either of the treatments, unpaired t-test with 95 % confidence interval was used.

### Safety analysis

The number and proportion of subjects with changes in laboratory value (change from baseline to end of study visit for complete blood count, serum creatinine, total bilirubin, urea, SGOT, SGPT, alkaline phosphates and calcium) was summarized and the difference was analyzed by chi –square test from normal to abnormal.

## Results

### Baseline characteristics:

In total, thirty eight individuals, thirteen healthy individuals with six control and thirteen patients with malabsorption syndrome with six control, fulfilled the eligibility criteria and completed the study (Fig. 1). Two participants (one healthy subject and one patient) reported vitamin D_3_ intake compliance of less than 90 % and therefore were not considered for final analyses.

In healthy subjects, at baseline, 85 % (n = 12) of the subjects had serum 25(OH)D concentration between 10 and 30 ng/ml, and one individual each (7.14 %) had serum 25(OH)D concentration >30 ng/ml and <10 ng/ml each in the oral soft gelatin capsule group. While in the buccal spray group, at baseline all subjects had serum 25(OH)D concentration between 10 and 30 ng/ml. Similarly, in patients with malabsorption disease who received oral soft gelatin capsules, at baseline 8 (57.14 %) patients had serum 25(OH)D concentration below 10 ng/ml, 5 (35.71 %) patients had serum 25(OH)D concentration between 10 and 20 ng/ml, and one individual (7.14 %) had serum 25(OH)D concentration above 20 ng/ml. However, in the buccal spray group, at baseline 9 (64.28 %) patients had serum 25(OH)D concentration below 10 ng/ml and 5 (35.71 %) patients had serum 25(OH)D concentration between 10 and 20 ng/ml. There were no striking differences in baseline characteristics between healthy individuals or patients with malabsorption disease with their corresponding control groups (Table 1).

**Table 1:** Demographic Data for Healthy Subjects and Patients with malabsorption syndrome

Parameters	Healthy Subjects Group I	Healthy Subjects Control Group II	Patients with malabsorption syndrome Group III	Patients with malabsorption syndrome Control Group IV
N	14	6	14	6
Sex	Male = 7, Female = 7	Male = 3, Female = 3	Male = 7, Female = 7	Male = 3, Female = 3
Age (Yrs)	36.21 ± 9.97	34.00 ± 6.42	39.93 ± 11.65	44.17 ± 5.56
(Range)	(25–60)	(25–42)	(26–63)	(38–53)
Height (cms)	159.86 ± 13.43	161.33 ± 14.12	162.29 ± 8.54	164.33 ± 8.55
BMI	23.39 ± 3.88	21.40 ± 2.39	21.48 ± 2.82	23.64 ± 3.02

All values are expressed in Mean ± SD; N-number subjects in each group

### Effect of intervention

**Fig. 2:**
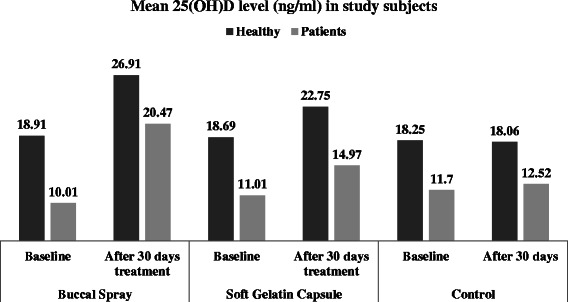
Mean 25(OH)D level in study subjects

The control groups in each subject population were compared with their corresponding treatment group. The control groups for healthy subjects as well as patients with malabsorption syndrome showed no change in 25(OH)D concentration over a period of 30 days. The mean baseline levels of 25(OH)D in healthy subjects was 18.25 ng/ml and in patients with malabsorption syndrome was 11.7 ng/ml. These mean levels remained at 18.06 ng/ml and 12.52 ng/ml after 30 days in healthy subjects and patients with malabsorption syndrome respectively (Fig. 2), which was statistically non-significant. When control group in healthy subjects was compared with their corresponding treatment groups, it was found that the difference of mean between control group and buccal spray group was 7.47 (95 % CI, 5.27, 9.67) which was significant (p < 0.05), and the same between control group and soft gelatin capsule group was 3.53 (95 % CI, 1.79, 5.28) which was also statistically significant (p < 0.05). Similarly in patients, the difference of mean between control group and buccal spray group was 8.53 (95 % CI, 2.74, 14.31) which was significant (p < 0.05), while the difference of mean between control group and soft gelatin capsule group was 2.03 (95 % CI, −1.44, 5.50) which was statistically not significant. This shows that buccal spray was more effective to increase mean 25(OH)D levels as compared to oral soft gelatin capsule. Table 2 describes these data.

**Table 2:** Comparison of 25(OH)D in control group and their corresponding treatment groups after 30 days

Comparison	Difference of mean	95 % CI	*P* Value
Healthy – Buccal Spray vs. Control	7.47	5.27 to 9.67	<0.0001
Healthy – Soft Gelatin Capsule vs. Control	3.53	1.79 to 5.28	0.0005
Patients – Buccal Spray vs. Control	8.53	2.74 to 14.31	0.0064
Patients – Soft Gelatin Capsule vs. Control	2.03	−1.44 to 5.50	0.2338

The efficacy of buccal spray and soft gelatin capsule to increase the 25(OH)D levels after 30 days of administration was evaluated and compared with each other. After 30 days of administration, overall mean serum 25(OH)D concentration in healthy subjects was 22.75 (sd 6.75) ng/ml as compared to baseline value of 18.69 (sd 5.88) ng/ml in the soft gelatin capsule group, with the mean increase of 4.06 (95 % CI 3.41, 4.71) ng/ml (Table 3). On the other hand, in the buccal spray group, the mean serum 25(OH)D concentration was 26.91 (sd 5.72) ng/ml as compared to baseline value of 18.91 (sd 4.3) ng/ml, with the mean increase of 8.0 (95 % CI 6.86, 9.13) ng/ml (Fig. 2). The difference in mean increase between both the groups was 3.95 (95 % CI 3.19, 4.69) which was statistically significant (p < 0.0001).

**Table 3:** Level of 25(OH)D in Healthy Subjects and Patients with malabsorption syndrome after 30 day administration of vitamin D3 buccal spray and soft gelatin capsule

Parameters	Mean baseline value in ng/ml	Mean value in ng/ml after 30 day of treatment	Difference of mean	Percentage mean increase from baseline
Healthy Subjects				
Soft gelatin Capsule Formulation				
Mean	18.69 ± 5.88	22.75 ± 6.75	4.06	21.72 %
Range	9.25, 30.05	12.75, 35.52	2.5, 5.6	15.36, 37.79
n	13	13	13	13
95 % CI	15.14 to 22.24	18.67 to 24.42	3.41 to 4.71	16.42 to 24.42
Buccal Spray Formulation				
Mean	18.91 ± 4.3	26.91 ± 5.72	7.995	42.99 %
Range	13.36, 26.82	19.6, 38.5	4.5, 11.67	29.21, 68.71
n	13	13	13	13
95 % CI	16.31 to 21.51	23.45 to 30.36	6.86 to 9.13	37.19 to 48.79
Patients with intestinal malabsorption syndrome				
Soft gelatin Capsule Formulation				
Mean	11.01 ± 6.43	14.97 ± 9.01	3.965	36.02 %
Range	2.9, 26.5	4.6, 36.89	1.06,10.39	24.62,58.73
n	13	13	13	13
95 % CI	7.12 to 14.89	9.52 to 20.42	2.37 to 5.56	30.42 to 41.62
Buccal Spray Formulation				
Mean	10.01 ± 4.29	20.47 ± 7.89	10.46	117.8 %
Range	4.6, 18.85	9.8, 34.64	4.25, 27.44	61.31,381.1
n	13	13	13	13
95 % CI	7.42 to 12.6	15.7 to 25.24	6.89 to 14.03	64.71 to 170.8

When calculated from baseline, the mean percentage change in serum 25(OH)D concentration in healthy subjects after 30 days treatment with soft gelatin capsule was 21.72 % (95 % CI 16.42, 24.42), while the same in buccal spray group was 42.99 % (95 % CI 37.19, 48.79) with a mean difference of 20.42 % (95 % CI 16.42, 24.42) between two groups (p < 0.0001) (Table 3). A total of 11 (85 %) subjects now had serum 25(OH)D concentration between 10 and 30 ng/ml, and two individuals (15.38 %) now had serum 25(OH)D concentration >30 ng/ml in the soft gelatin capsule group. However, in the buccal spray group, 10 (75.88 %) subjects had serum 25(OH)D concentration between 10 and 30 ng/ml and 3 (23.1 %) subjects now had serum 25(OH)D concentration >30 ng/ml.

Similarly in patients with malabsorption syndrome, overall mean serum 25(OH)D concentration after 30 days of administration of soft gelatin capsule was 14.97 (sd 9.01) ng/ml as compared to baseline value of 11.01 (sd 6.43) ng/ml, with the mean increase of 3.96 (95 % CI 2.37, 5.56) ng/ml. While in buccal spray group, mean serum 25(OH)D concentration was 20.47 (sd 7.89) ng/ml as compared to baseline value of 10.01 (sd 4.29) ng/ml, with the mean increase of 10.46(95 % CI 6.89, 14.03) ng/ml (Fig. 2). The difference in mean increase between both the groups was 6.50 (95 % CI 3.78, 9.22) which was statistically significant (p < 0.0001).

The mean percentage change in serum 25(OH)D concentration in patients with malabsorption syndrome after 30 days treatment with soft gelatin capsule was 36.02 % (95 % CI 30.42, 41.62), while the same value in buccal spray group was 117.8 % (95 % CI 64.71, 170.8) with a mean difference of 81.75 % (95 % CI 29.80, 133.7) between the two treatments (p < 0.005). A total of four (31 %) subjects now had serum 25(OH)D concentration below 10 ng/ml, eight (61 %) subjects now had serum 25(OH)D concentration between 10 and 30 ng/ml, and one individual (7.7 %) had serum 25(OH)D concentration above 30 ng/ml. However, in the buccal spray group, only 7.7 % (1 subject) now had serum 25(OH)D concentration below 10 ng/ml, ten (76.9 %) subjects now had serum 25(OH)D concentration between 10 and 30 ng/ml and two (15.4 %) subjects now had serum 25(OH)D concentration more than 30 ng/ml (Table 3).

### Statistical considerations with respect to period, sequence and power:

Statistical analyses were performed using SAS v9.2 (SAS Institute Inc, Cary, NC, USA) for the post-hoc evaluation of sequence and period effect. The statistical method adopted for this analysis was period + sequence + Subject (sequence) + treatment. It was observed that there is no significant difference in sequence effect in healthy subjects (p = 0.5251) and in patients with malabsorption syndrome (p = 0.0532). It was also revealed that there is no statistically significant period effect in healthy subjects (p = 0.6920) as well as in patients with malabsorption syndrome (p = 0.0715). The post-hoc power analyses with intra-subject variability were also derived for both the group of subjects. In healthy subjects, statistical power obtained was 99.42 with an 11.81 % intra-subject variability. While, in patients with malabsorption syndrome it was 81.62 with an intra-subject variability of 21.86 %.

### Safety evaluation

There were no significant changes in any of the hematology and biochemistry parameters studied. There are also no notable changes in the vitals for any of the participants. No adverse event reported after administration of the buccal spray or soft gelatin capsule formulations of vitamin D_3_ during entire study period in healthy or patients with malabsorption disease and hence the product is considered safe.

## Discussion

Supplemental fat-soluble vitamin D is usually made without determination of whether oral doses are adequately absorbed. The evidence of vitamin D malabsorption (Osteomalacia, rickets, hypocalcaemia, or reduced circulating concentration of 25(OH)D) persists despite routine vitamin D supplementation in cystic fibrosis [14], Crohn’s disease [15], Intestinal resection [16–19], ulcerative colitis, liver disease [20–22] and other malabsorption syndrome [23].

Many factors are involved in the absorption of vitamin D, including gastric, pancreatic, and biliary secretions, micelle formation, and diffusion through the unstirred water layer, brush border membrane uptake, and transport out of the intestinal cell [24, 25]. As vitamin D is a relatively non-polar sterol, it must be solubilized by incorporation into a bile salt micelle solution in order to be absorbed in the aqueous phase [26]. This process is severely inhibited if there is any interruption of normal pancreatic or biliary secretion. As fat-soluble vitamins are fairly sensitive to disturbances in lipid absorption, vitamin malabsorption may occur in conditions like steatorrhea, ulcerative colitis, and Crohn’s disease. Serum concentrations of 25-hydroxyvitamin D are good indicators of long term vitamin D levels in the body but are insensitive to single doses of vitamin D and do not rise out of the normal range unless doses of vitamin D are chronically administered [27].

Considering the malabsorption in intestinal disease and a possible poor and complex absorption with oral formulations, a novel nanoemulsion formulation of buccal spray was developed where vitamin D_3_ is suspended in an aqueous base which can be easily absorbed through the mucosal layer of the mouth. We compared the serum concentration of vitamin D after 30 days administration with the soft gelatin capsule and the aqueous based buccal spray formulation. To the best of our knowledge, this is the first randomized two way cross over trial comparing the increase in 25(OH)D levels in healthy adults and patients with intestinal malabsorption receiving similar oral doses of two different formulations (per oral and buccal spray).

The analyses of baseline levels of study participants showed that vitamin D deficiency was prevalent in both healthy subjects and patients with intestinal malabsorption syndrome. However, patients were more vitamin D deficient as compared to healthy subjects. Four weeks administration of 1000 IU per day increased mean serum 25(OH)D in all treatment groups. In healthy subjects, soft gelatin capsule increased serum 25(OH)D level by 22.5 % (Range 15.4 to 37.8 %), while the buccal spray increased serum 25(OH)D level by 43 % (range 29.2 to 68.7. Similarly, in patients with intestinal malabsorption, soft gelatin capsule increased serum 25(OH)D level by 36 % (range 24.6 to 58.7 %) and the buccal spray increased serum 25(OH)D by 117.8 % (range 61.3 to 381.1 %).

The result implies that the buccal spray formulation had a significantly higher mean increase in both the subject groups, healthy subjects and patients with intestinal malabsorption syndrome. Interestingly, the mean increase was much higher in the patients group as compared to the healthy subjects group. This may be because increase in serum 25(OH)D after supplementation is known to be inversely related to baseline 25(OH)D concentration [28].

In the present study, the mean baseline levels were almost half in patients with intestinal malabsorption as compared to the baseline levels found in healthy subjects. This indicates the presence of vitamin D_3_ deficiency in the patient group as compared to healthy subjects. The primary and the most important source of vitamin D is sunlight. Although excessive exposure to sunlight and vitamin D have been positively associated with non-melanoma skin cancer [29], ecological studies suggest that sunlight may protect against female breast, ovarian, prostate, and colon cancer [30]. Solar UV-B exposure and the amount of exposure to sun are related inversely with cancer mortality and survival in detailed epidemiological studies [31]. Some analytical studies suggest a protective association between circulating vitamin D in blood, which is largely derived from sunlight or dietary vitamin D, and colorectal cancer and prostate cancer [30]. However, looking at the overall baseline levels of all participants in the present study, it is also advisable to increase the moderate daily sun exposure and to improve clothing apart from vitamin D_3_ supplementation.

## Conclusion

We conclude that the buccal spray formulation was able to increase mean serum vitamin D_3_ concentration significantly higher as compared to the soft gelatin capsule, in both healthy subjects (1.9 times) as well as in patients with intestinal malabsorption syndrome (2.6 times).

## Availability of supporting data

The data set(s) supporting the results of this article is (are) included within the article.

## Abbreviations

25(OH)D: 25-hydroxyvitamin D
CTRI: clinical trial registry of India
BMI: Body Mass Index
ECLIA: Electrochemiluminescence
IU: International Unit
